# Gold—No. VI

**Published:** 1859-08

**Authors:** 


### GOLD.—Number VI.

BY PROF. AUSTIN.

*Gold of absolute purity is used for comparatively few purposes. The best gold leaf, dentist's foil, solder for platina work, and the positive plate of an electro-gilding apparatus should be as pure as possible. The most of the gold leaf

* Eratum.—In last No. 46th page, 17 lines from the bottom, for 108.2 read 103.2.

used in the arts contains a small proportion of alloy, the effects of which will be subsequently considered. Dentist's foil also usually contains some alloy, but the best manufacturers at present aim to give to their foil the highest attainable degree of purity. A small quantity of alloy, about six grains to the oz., aids materially in giving to the foil the property of non-adhesiveness, required for certain methods of operating. The opposite quality of adhesiveness, is most fully developed in the purest metal. But it is also true that this property of gold is very much under the control of the gold beater: since from the same ingot he can produce, at will, adhesive or non-adhesive foil. So small a proportion of alloy, (1-80) of silver or copper can scarcely do injury to the teeth or mouth; but it gives a liability to tarnish, which, if occurring on a surface in a contact with a thin shell of enamel, produces an unsightly discoloration. The writer has seen foil discolor in the mouth and even in the process of annealing, which was asserted by a perfectly honest manufacturer to be as absolutely pure as could be made. Whether there was in such cases some accidental unsuspected impurity, some minute speck of copper affecting the metal around it, and when hammered out spreading its effects over considerable surface; or whether the iron upon which the annealing process is conducted can in any way have an effect upon the foil —are questions which we suggest but cannot answer.

Pure gold is used by dentists in a sponge-like form known as sponge-gold or crystal-gold. It is not in place here to enter at all into the relative merits of crystal gold and foil; both have their warm advocates. In the preparation of the sponge or crystal gold it is of the utmost importance that every trace of aqua regia and mecury, when one or both are used in the manufacture, should be removed. When this form of gold was first introduced this important cleansing process was imperfectly done; but subsequent improvements have quite removed this objection to its use.

The adhesive quality of gold leaf or foil is due to the highly developed cohesive attraction of the particles. This force is diminished by the presence of any alloy and also by exposure to the air after annealing, and is quite destroyed by the presence of moisture. Simple contact will inseperably unite two leaves of adhesive foil: but in uniting a number of layers together moderate pressure is requisite. A piece of gold carefully formed in this way, has the same physical characteristics as if it had been solidified by melting: its perfect cohesion throughout can be most severely tested by drawing it out into fine wire—an experiment which has often been successfully performed. In a similar manner two halves of a freshly cut lead bullet will firmly reunite upon pressure: two surfaces of recently prepared plate glass will frequently unite inseperably if placed for some time in contact. In two metals, iron and platina, this cohesive force is developed only at a very high temperature, and under heavy pressure. This well known quality of welding gives a greatly increased value to iron. To the analogous but less known property of gold has been given the name of *cold welding.*

The preparation of gold leaf forms a large business to itself, requiring a great deal of care and experience. Gold is by far the most laminable or malleable of metals. It may be made so thin that 367,500 leaves are required to make an inch in thickness. The same number of leaves of ordinary writing paper would form a column 100 feet high: in other word it would require 1200 leaves of such foil to make the thickness of a sheet of writing paper. A sheet of No. 4 dentists' foil would make from 25 to 30 such leaves. But it is only the purest gold that admits of such extreme attenuation. Practically it is not carried so far, because the leaves would be too thin for convenient handling and a little alloy is added; 290,000 to the inch is the usual thickness. Some gold beaters will tell you that they must necessarily use pure gold, because any alloy lessens its maleability. This is true since silver, second in this respect to gold, can be hammered only as thin as the 10,000th part of an inch. But there are few gold beaters who do not use from 6 to 16 grains alloy to the ounce. This amount will prevent troublesome adhesion of the leaves to one another, and thus far it is very useful, and also allows the leaf to be reduced to the standard thinness used in the arts. Undoubtedly it gives to the leaf some liability to tarnish; but the gold leaf used in the arts which changes color so rapidly will be found still more highly alloyed. This is a fraudulent practice, done for the sake of economy, each grain of alloy saving to the manufacturer between four and five cents.

Any desired shade, within certain limits, may be given the leaf. The red color is given by copper, from 12 to 16 grains per ounce: orange and lemon colors by 6 to 8 grains of copper mixed with from 12 to 20 grains of silver: greenish and white shades by silver alone, from 2 to 20 pennyweights.—Copper hardens and impairs the malleability of the gold much more than silver: and when either metal is present in large proportion, the addition of a very small quanty of the other, materially adds to this effect. For this reason, in red alloys with copper and in green and white alloy with silver, only one metal must be used.

The art of gold beating has been known and practiced for more than three thousand years. The Jews were familiar with it and the Egyptians had brought it to a high state of perfection: still it was about three times as thick as the leaf of modern times.

The gold, alloyed as required, or else pure, is melted at a higher temperature than is necessary for simple fusion, which gives it greater malleability, and cast into ingots 1½ inches long, ¾ inch wide and 3-16ths inch deep. Annealing in hot ashes removes the grease of the ingot mould, and also increases its malleability. The French workmen next forge out the ingot, hammering it down to one-sixth of an inch, with repeated annealings. But the English gold-beaters proceed at once to the process of *lamination* between rollers, which for this purpose require to run with great accuracy. An ounce of gold thus treated forms a riband some ten feet long, an inch and a half wide, and about 1-800th inch thick. This strip is rolled up and thoroughly annealed, then cut very exactly into pieces about an inch or an inch and a quarter square, weighing 6 or 7 grains. One hundred and sixty of these squares are next placed between the leaves of a *Cutch*, made of vellum, or of a very tough paper, manufactured expressly for the purpose.

The gold-beater's art is answerable for the loss of many a precious manuscript—parchments from which, in preparing them for the ignoble purpose of a cutch, were erased characters that to the student would have given them a value above all the gold ever placed between their leaves. Thoughts of great minds, lost records of noble deeds, important events in the world's history, all sacrificed to the "yellow god"—one instance out of a thousand showing how often we unconsciously sacrifice the greater to the lesser good. Modern art has quite recently made from paper a very remarkable artificial parchment by passing it through a sulphuric acid bath, the strength of which requires the nicest regulation. This will probably supersede the use of parchments, though it can not recall the valuable writings which, because of the superior quality of the material on which they were written, have been sacrificed.

The cutch has two strong cases of parchment drawn over it at right angles to each other, so that the sides of one cover the open ends of the other. It is then beaten with a 17 pound hammer upon a firmly placed block of marble. The package is frequently bent or rolled with the hands, to prevent the leaves adhering to the vellum, and is occasionally opened to shift the inner leaves to the outside, and *vice versa*, to equalize the force of the hammer. In about a half hour each of the inch square plates will be beaten out to four square inches. These are taken out, cut into inch square pieces, and placed between the leaves of another *tool* called a *shoder*, which is made of gold-beaters' skin.

Gold-beaters' skin is prepared from the cœcum of the ox. The membranes are well prepared in an alkaline solution. One membrane is then stretched in a frame and another laid upon it. The mucous surfaces being brought together, unite readily and firmly, thus giving to each skin a smooth serous surface on both sides. This double membrane is then treated with alum and some albuminous substance, then beaten between layers of paper to remove the grease, and afterwards preserved and dried. The details of the process are usually conducted with much secrecy. The English excel in the manufacture of this article, which requires great care and commands a high price. A package of skins is called a *mould*, containing in Europe 1,200 leaves, in England and America 850 leaves. An English mould, containing 850 double leaves, a little over five inches square, requires for its production the slaughter of 500 oxen. After several months' use the skins lose their flexibility, which is in a great measure restored, by placing between leaves of paper and moistening them with white wine; after being pressed for three or four hours, they are put between parchment leaves and beaten for a day, then rubbed with fine calcined plaster. Dampness is very injurious to them.

The *shoder* of skins, interleaved with the gold squares, is enclosed in a double parchment case, as was the cutch, and beaten about two hours with a ten or twelve-pound hammer. During this and the subsequent hammering, the packet must be repeatedly rolled in the hand, to loosen the gold and prevent its adhering to the skins.

The leaves are then removed and each one subdivided into four pieces, by means of two sharp-edged pieces of cane, set at right angles upon a small board: a steel knife is not fit to cut the leaf when very thin, because of its liability to adhere to the metal.

These squares are placed in another *shoder*, composed of the finest and most perfect skins, and beaten for four hours with a seven-pound hammer. This last process requires great dexterity, on the part of the workman, to secure uniformity in the now extremely attenuated leaf. Three beatings and two quarterings expand the gold nearly 200 times more than when in the form of a riband, one ounce in this state covering 100 square feet. It could be still further expanded to cover 160 square feet, but this would involve too much extra trouble in the process of beating, and the leaves would be very difficult to handle. The leaves are carefully removed with long delicate wooden pincers, flattened by the breath upon a leather cushion, and cut with a frame of sharp cane into squares of uniform size, then placed in small paper books, the leaves of which are rubbed with red ochre to prevent adhesion. Dentists' foil, not being so thin, is placed in books of white laid paper, larger in size than a gilder's book, and requiring no ochre. A guilder's book contains 25 leaves, while a book of foil for dental use contains $\frac{1}{8}$ or $\frac{1}{4}$ oz.—each sheet weighing from 4 to 10 grains, and sometimes as high as 20 grains. The properties of the foil or leaf are much affected by the manner of annealing. In fact, there are several nice points in the manufacture of dentists' foil and gilders' leaf, about which gold-beaters are not particularly communicative; for to their skill in some one or more of them they attribute the peculiar excellence of their own article.

The labor of gold-beating is arduous, and attempts have of late been made to supersede manual labor by machinery. Eventually the majority of the work may be thus done, but skilled hand work will always, we apprehend, be in demand for the best quality of gold leaf.

